# The Ability of Virulence Factor Expression by *Pseudomonas aeruginosa* to Predict Clinical Disease in Hospitalized Patients

**DOI:** 10.1371/journal.pone.0049578

**Published:** 2012-11-12

**Authors:** Michel Ledizet, Thomas S. Murray, Sailaja Puttagunta, Martin D. Slade, Vincent J. Quagliarello, Barbara I. Kazmierczak

**Affiliations:** 1 L2 Diagnostics, New Haven, Connecticut, United States of America; 2 Department of Pediatrics (Infectious Diseases), Yale University School of Medicine, New Haven, Connecticut, United States of America; 3 Department of Medicine, Sections of Infectious Diseases, Yale University School of Medicine, New Haven, Connecticut, United States of America; 4 Department of Occupational & Environmental Medicine, Yale University School of Medicine, New Haven, Connecticut, United States of America; 5 Department of Microbial Pathogenesis, Yale University School of Medicine, New Haven, Connecticut, United States of America; The Scripps Research Institute and Sorrento Therapeutics, Inc., United States of America

## Abstract

**Background:**

*Pseudomonas aeruginosa* is an opportunistic pathogen that frequently causes hospital acquired colonization and infection. Accurate identification of host and bacterial factors associated with infection could aid treatment decisions for patients with *P. aeruginosa* cultured from clinical sites.

**Methods:**

We identified a prospective cohort of 248 hospitalized patients with positive *P. aeruginosa* cultures. Clinical data were analyzed to determine whether an individual met predefined criteria for infection versus colonization. *P. aeruginosa* isolates were tested for the expression of multiple phenotypes previously associated with virulence in animal models and humans. Logistic regression models were constructed to determine the degree of association between host and bacterial factors with *P. aeruginosa* infection of the bloodstream, lung, soft tissue and urinary tract.

**Results:**

One host factor (i.e. diabetes mellitus), and one bacterial factor, a Type 3 secretion system positive phenotype, were significantly associated with *P. aeruginosa* infection in our cohort. Subgroup analysis of patients with *P. aeruginosa* isolated from the urinary tract revealed that the presence of a urinary tract catheter or stent was an additional factor for *P. aeruginosa* infection.

**Conclusions:**

Among hospitalized patients with culture-documented *P. aeruginosa,* infection is more likely to be present in those with diabetes mellitus and those harboring a Type 3 secretion positive bacterial strain.

## Introduction

Hospital acquired infections (HAI) are estimated to complicate 5–10% of hospitalizations in the United States annually, leading to increased health care costs and prolonged hospitalizations [1]. *Pseudomonas aeruginosa* is a frequent cause of HAI, isolated in 16% of urinary tract infections and 18% of pneumonias, particularly ventilator-associated pneumonia [2], [3]. A combination of several factors–intrinsic antibiotic and microbicide resistance, prevalence and persistence in the hospital environment, and a propensity to form biofilms on medical devices–lead to relatively high colonization rates by this organism. Prospective studies have shown that a subgroup of colonized patients develop clinically significant disease, such as ventilator-associated pneumonia. However, many patients do not progress to invasive disease, suggesting that differences in bacterial virulence and/or host susceptibility influence clinical course [4]–[7].

Many *P. aeruginosa* virulence factors have been identified over the past decades. The most robust virulence factor in animal models and human studies is the Type 3 secretion system (T3SS), a specialized protein secretion apparatus that allows Gram-negative bacteria such as *P. aeruginosa* to translocate a specific subset of bacterial effector proteins into the host cell cytosol [8]. Only four *P. aeruginosa* effectors have been identified to date: Exotoxin Y (ExoY), an adenylate cyclase; ExoT and Exo S, related proteins that exhibit both GTPase activating activity and ADP-ribosyl transferase activity; and ExoU, a phospolipase A_2_ [9]–[15]. Carefully controlled studies examining virulence of isogenic T3SS mutants in murine models of acute pneumonia have demonstrated that two secreted effectors, ExoU and ExoS, are independently associated with increased virulence in this model. Other virulence factors identified in animal models include those promoting bacterial motility and adhesion (i.e., flagella and Type IV pili) [16]–[21], degradative enzymes [22]–[25], and genomic pathogenicity islands [26]–[31].

Bacterial expression of the T3SS is co-regulated with expression of many bacterial traits associated with acute infection (flagella, type IV pili, secreted proteases) or chronic colonization (biofilm formation). As inverse regulation of acute vs. chronic virulence factors is a feature of many of these regulatory networks, some authors have postulated that bacteria may switch their behavior to favor acute infection or chronic colonization [32]. The natural history of *P. aeruginosa* in Cystic Fibrosis patients is thought to illustrate such a switch, as younger CF patients are usually acutely infected by T3SS-positive strains, but progress to long-term colonization by T3SS-negative strains that usually display mucoid or hyper-biofilm phenotypes [33], [34]. No study has asked, however, whether bacterial expression of particular virulence factors is associated with infection vs colonization in non-CF patients. Identifying such associations might have significant utility for clinical decision-making for hospitalized patients with positive *P. aeruginosa* cultures. Discriminating between patients at high vs. low risk of acute infection could improve delivery of effective anti-pseudomonal therapy to infected patients, but decrease inappropriate antibiotic use in colonized patients.

In this study, we prospectively enrolled 248 unique hospitalized patients with *P. aeruginosa* cultured from blood, airway secretions, urine, or deep wound specimens, excluding individuals with Cystic Fibrosis. Subjects were followed clinically for five days after their positive culture to determine whether they met pre-determined clinical criteria for infection vs. colonization with *P. aeruginosa*. Selected bacterial phenotypes associated in the literature with virulence or colonization were assayed for each bacterial isolate. We employed the statistical tool of factor analysis to determine whether any of the measured bacterial variables grouped together in orthogonal “factors” [35]. The primary endpoint of our study was to determine which, if any, readily measured bacterial traits were associated with *P. aeruginosa* infection versus colonization of the respiratory or urinary tract.

## Methods

### Cohort Assembly

This study was approved by the Institutional Review Board of Yale University School of Medicine (HIC #0411027243). All pediatric and adult patients hospitalized at Yale New Haven Hospital (New Haven, CT) with a positive culture for *P. aeruginosa* from the respiratory tract, urinary tract, blood, or deep tissue were eligible for study enrollment if they, or designated proxies, provided written consent. Clinical isolates were identified as *P. aeruginosa* if they grew as non-lactose fermenting colonies on MacConkey agar, were oxidase positive, could utilize acetamide as a sole carbon source, and gave a characteristic zone of inhibition to colistin on Kirby-Bauer plates. The presence of characteristic “grape-like” odor, pigment production and ability to grow at 42°C were also noted. Isolates from enrolled patients were subcultured from either primary specimen plates (i.e. blood or MacConkey agar) or from Kirby-Bauer plates generated by the Clinical Microbiology laboratory to Luria Broth (LB) agar, then coded and banked as 15% glycerol stocks at –80°C. Patient demographics, risk factors for infection, co-morbid medical conditions, prior and concurrent antibiotic exposure, history of prior *P. aeruginosa* cultures, clinical and laboratory features were collected at baseline and for 5 days after the initial culture date. Patients were excluded from the study if they had Cystic Fibrosis (n = 5), had *P. aeruginosa* isolates that would not grow in the defined media required for the Type 3 phenotypic assay (n = 2), or had isolates cultured from a non-eligible site (i.e. a biliary catheter drainage bag) (n = 1).

### Clinical Outcomes

Anonymously coded data collection forms were reviewed independently by two Infectious Diseases physicians to determine whether patients met criteria for infection of the respiratory tract (pneumonia), urinary tract (cystitis or pyelonephritis) or soft tissue/bone (abscess or osteomyelitis), based on definitions established by the National Nosocomial Infection Surveillance (NNIS) system (Table 1) [36]; reviewing physicians were blinded to virulence testing results. Patients who met clinical criteria were categorized as infected; those who did not were categorized as colonized. Bloodstream isolates were considered indicative of infection. If the patient met the criteria for infection, but other potential pathogens were co-cultured with *P. aeruginosa*, the patient was categorized as “infection, possibly due to *Pseudomonas*”. Potential pathogens were considered to be: (1) any other organism isolated from blood or an intraoperative tissue culture, (2) any organism present in >10^5^ cfu/ml in urine, or (3) *Staphylococcus aureus, Streptococcus pneumoniae, Hemophilus influenzae, Moraxella catarhalis*, any Gram-negative rod not normally isolated from the oropharynx, *Mycobacterium tuberculosis*, and dimorphic fungi or molds in respiratory tract specimens. The report of “normal respiratory flora” without the specific identification of one of these microorganisms, or the isolation of *Candida albicans* in sputum, was not interpreted as indicating the presence of another potential pathogen. When the two reviewers disagreed, a third independent review was performed to determine clinical assignment of infection versus colonization.

**Table 1:** Clinical criteria for infection based on site of culture.

**Respiratory tract:** **Infection (pneumonia)**	*P. aeruginosa* cultured from respiratory specimen containing fewer than 25 epithelial cells/HPF.
	New and persistent infiltrates are noted on chest x-ray or computed tomography (CT).
	Two of the following three criteria are met: (1) fever >38.3°C; (2) WBC>12,000 or bands/metamyelocytes ≥6% or1500/µl; (3) altered mental status (patient >70 y).
	Symptoms referable to the respiratory tract, evidenced by at least one of the following: (1) new purulent sputum ORincreased sputum, secretions or suctioning; (2) new/worsening cough OR dyspnea OR tachypnea; (3) worsening gas exchange(increased requirement for supplemental oxygen, increased ventilator support, PaO_2_/FiO_2_≤240).
**Urinary tract: Infection** **(cystitis or** **pyelonephritis &** **urosepsis)**	Greater than 10^5^ cfu *P. aeruginosa*/ml urine
	Pyuria (>10 WBC/HPF)
**Soft tissue/bone:** **Infection (abscess or** **osteomyelitis)**	*P. aeruginosa* cultured from deep wound or intraoperative tissue specimen
	Clinical evidence of infection (purulent drainage, pain/erythema/dehiscence) or histopathological confirmation of infection

### Testing of Type III Protein Secretion by ELISA

Components of the T3SS were detected quantitatively by an ELISA assay as described previously [37]. Monoclonal antibodies against ExoS (SD1, SE1) were used to detect ExoS simultaneously with the other antigens.

### Isolation of Monoclonal Antibodies Against ExoS

Monoclonal antibodies against ExoS were isolated using techniques described in detail previously [37]. Briefly, ExoS was partially purified from culture supernatants of PA103 Δ*exoU* Δ*exoT attB*::*exoS*. Splenocytes were isolated from immunized mice and fused to myeloma cells. Antibody-producing cell lines were identified by performing ELISAs on hybridoma culture supernatants. Two monoclonal antibodies, SD1 and SE1, were selected for the immunoassay described here. The specificity of the antibodies was ascertained by demonstrating that they reacted in ELISAs against culture supernatants of PA103 Δ*exoU* Δ*exoT attB::exoS* and PAO1, but not PA103 Δ*exoU* Δ*exoT*, PA14 or PA103. In immunoblots, both antibodies recognized a single protein with a molecular weight of 48,000, consistent with the known structure of ExoS. Isotyping of the purified immunoglobulins was performed with a kit (Pierce) according to the manufacturer’s instructions. Both SD1 and SE1 are IgG1 and contain a kappa light chain. Competition experiments showed that SD1 and SE1 recognize different epitopes on the ExoS molecule.

### Motility Assays

Swimming, twitching and swarming motility were measured as previously described [38]. Each isolate was independently assayed twice.

### Static Biofilm Formation

Static biofilm formation on polystyrene was measured in triplicate using the method of O’Toole [39].

### Secreted Protease Activity

Protease production was measured by a casein proteolysis microplate assay as previously described [38]. The LasB elastase, alkaline protease, and protease IV are all capable of degrading casein [40].

### Pathogenicity Island Loci

Genomic DNA was amplified by PCR using primers specific for PAPI-I pathogenicity island loci RL033 (RL033F: 5′ AAAGTTTAGCGCCGACTTCA; RL033R: 5′ GAAGTGAGTTGGGCACCATT) or RL112 (RL112F: 5′ GACTGGAATAGACGCCCAAG; RL112R: 5′AAACCTTCGACGGTGATCTG). This pathogenicity island was interrogated based on its association with virulence in several distinct animal models of virulence; the queried loci encode two experimentally identified “pathogenicity genes” [26], [31].

### Statistical Analysis

Values obtained for all measured bacterial virulence phenotypes were subjected to an exploratory factor analysis [35]. The principal factor method was used to extract factors, followed by promax (oblique) rotation. A “scree” test was used to determine the number of meaningful factors retained for rotation. An item was considered to load on a given factor if the factor loading was 0.50 or greater for that factor and was less than 0.50 for all other factors.

Unadjusted and adjusted logistic regression models were conducted. Parsimonious models were obtained through the use of a forward selection strategy with a significance-to-enter defined as 0.06. The outcome was defined as infection or sepsis caused by *P. aeruginosa* vs. colonization. Patients in whom infection was present but could not be attributed to *P. aeruginosa* (i.e. another potential pathogen was co-cultured) were coded as unknown and excluded from our analysis. All statistical analyses were performed using SAS 9.1.

## Results

### Patient Characteristics

A total of 248 patients with *P. aeruginosa* cultured from blood, urine, respiratory secretions or tissue provided written informed consent and were enrolled in this study at Yale New Haven Hospital (YNHH) from July 2005 through March 2007. Eight patients were found to be ineligible based on defined exclusion criteria. One eligible *P. aeruginosa* isolate was discarded by the Clinical Microbiology laboratory. Two additional individuals were enrolled twice, during independent hospitalizations under different medical record numbers; the second enrollment event was not analyzed in this study, resulting in a total of 237 unique participants.

Patient age ranged between <1 to 96 years at the time of enrollment (mean, 58.6 years). One hundred sixty (68%) patients were admitted to YNHH from home, while 48 (20%) were admitted from rehabilitation or extended care facilities. Twenty-six (11%) patients were transferred from other hospitals, while three patients remained at YNHH since their birth. The median elapsed time between hospital admission and the positive *P. aeruginosa* culture that triggered enrollment was 4 days (range, 0 to 1749); for 79 patients (33%), a prior positive culture for *P. aeruginosa* was documented during either the index or a previous hospitalization.

Host factors historically associated with increased risk of *P. aeruginosa* infection were assessed in our cohort [41]. Sixty-five (27%) patients carried a diagnosis of Type I or Type II diabetes mellitus. Fifty-five (23%) patients were deemed immunocompromised as a result of chronic steroid use (≥10 mg prednisone per day for 30 days or longer), post-transplant immunosuppression, marrow suppressive chemotherapy or HIV/AIDS. Seventy-two patients (30%) carried a diagnosis of asthma, chronic obstructive pulmonary disease or emphysema, or had radiologic evidence of bronchiectasis; these individuals were considered to have “chronic lung disease” in our analysis. The majority of patients (183/237; 77%) had at least one medical device that might be expected to predispose to infection of the respiratory tract (i.e., endotracheal tube or tracheostomy), urinary tract (i.e., bladder catheter or nephrostomy tube) or blood (i.e., central intravascular catheter). In 144 patients (61%) such a device was present at the anatomical site from which *P. aeruginosa* was cultured, and was considered a “relevant foreign device” in our analysis.

### Clinical Characteristics of Patients

Seventy-one patients (30%) met the criteria for infection of bloodstream, respiratory tract, urinary tract or soft tissue/bone, and had no other potential pathogens cultured from these sites except for *P. aeruginosa*. An additional 52 patients (22%) met criteria for infection, but at least one other potential pathogen was cultured from the site of infection in addition to *P. aeruginosa*. One hundred fourteen patients (48%) did not meet the criteria for infection despite a positive culture for *P. aeruginosa* and were classified as “colonized”. This group included 65 individuals with pure *P. aeruginosa* cultures (urine or wound) or normal respiratory flora plus *P. aeruginosa*, and 49 patients whose cultures yielded a potential pathogen in addition to *P. aeruginosa*. Table 2 shows the distribution of patient age, gender, ethnicity and selected comorbidities among the groups.

**Table 2:** Patient characteristics as a function of colonization vs. infection status.

	Colonization (n = 114)	*P. aeruginosa* Infection/Sepsis (n = 71)	Infection, possibly due to*P. aeruginosa* (n = 52)	*p* value (chi square)
Gender	51 (45%) female	24 (34%) female	24 (46%) female	0.26
Mean age, yrs (95% CI)	58.0 (53.3–62.7)	61.4 (56.4–66.4)	58.3 (51.4–65.2)	–
Ethnicity (Hispanic)	21 Hispanic (18%)	5 Hispanic (7.0%)	5 Hispanic (9.6%)	0.06
Diabetes	26 (23%)	27 (38%)	12 (23%)	0.06
Immunocompromised	31 (27%)	17 (24%)	17 (33%)	0.56
Relevant foreign device	67 (59%)	46 (65%)	31 (60%)	0.70

### Bacterial Isolates

We collected sputum isolates from 136 patients, urine isolates from 65 patients, and deep wound/tissue isolates from 22 patients. Ten patients had blood isolates; in addition, one patient had positive cultures from both sputum and blood, two from both urine and blood, and one from wound and blood. Table 3 shows the distribution of clinical outcomes by site of culture. Each bacterial isolate was assayed for each virulence phenotype as described in Methods. For the first 75 individuals enrolled in the study, we collected 10 independent *P. aeruginosa* colonies from the primary culture plates and assayed each individually. Although heterogeneity of T3SS phenotype expression has been reported among isolates cultured from patients with CF [34], we found that only 4 (5.3%) of our tested samples harbored mixtures of T3SS-positive and –negative strains. Given this low rate of T3SS phenotype heterogeneity, only one isolate per unique anatomic site was collected and phenotyped in subsequently enrolled patients.

**Table 3:** Sources of bacterial isolates as a function of clinical categorization.

Site	Colonization(n = 114)	*P. aeruginosa* Infection/Sepsis(n = 71)	Infection, possibly due to *P. aeruginosa*(n = 52)
Respiratory	80	25*	32
Urine	30	29*	8
Soft tissue/bone	4	9*	10
Blood		12	2

* Patients with *P. aeruginosa* isolated concurrently from blood and another site (n = 4) are analyzed with both blood and non-blood sites.

A number of the bacterial variables that we measured in our study are likely to be biologically interrelated. We applied the statistical tool of factor analysis to identify which variables were most highly interdependent, and found that most of the bacterial phenotypes loaded into three mathematically defined factors [35]. Factor 1 (“Type 3 secretion”) reflected secretion of the two T3SS structural proteins, PopD and PcrV, and of the T3SS substrates ExoT, ExoS and ExoU. Factor 2 (“Flagellar motility & proteases”) consisted of two flagellar based forms of motility (swimming and swarming) and protease production. Factor 3 (“Fimbriae”) consisted of twitching motility and the presence of two loci, RL033 and RL112, within the PAPI-1 pathogenicity island. Equations for factors 1, 2 and 3 are listed in Figure S1. Biofilm formation did not load strongly onto any of the three factors and was thus evaluated as an independent variable in our models.

### Bacterial and Host Variables Associated with Increased Risk of Infection

We tested the measured bacterial phenotypes, as well as host variables potentially associated with altered risk of infection, in both an unadjusted (bivariate) logistic regression model and a parsimonious model resulting from multivariate logistic regression models using a forward selection strategy. All tested model variables are listed in Table 4. Patients with infections that could not be definitely attributed to *P. aeruginosa* because another potential pathogen was co-cultured were considered to have an “unknown” clinical categorization, and their records were excluded from analysis, lowering the sample size from 237 to 185. Results of the parsimonious (forward selection) model are presented in Table 4. Only two variables, diabetes mellitus and “Type 3 secretion” (Factor 1) showed a significant association with infection vs. colonization (p≤0.05). We also ran our models on the subsets of patients with positive cultures from the respiratory tract (n = 105) or urine (n = 59). In patients with positive respiratory specimens, no host or bacterial variable was significantly associated with patient outcome (infection vs. colonization) in the multivariate analysis, and no variables entered the parsimonious model. In patients with positive urine cultures, two independent parsimonious models could be constructed. Both contained diabetes mellitus and the presence of a urinary catheter or stent as host variables significantly associated with increased risk of infection, plus a bacterial variable which was either “Flagellar motility & proteases” (odds ratio 2.3; p = 0.02) or “Type 3 secretion” (odds ratio 1.9; p = 0.05).

**Table 4:** Parsimonious multivariate analysis of factors associated with risk of *P. aeruginosa* infection.

Parameter	Level	Parsimonious (forward selection), All culture sites					Parsimonious (forward selection), Urinary tract cultures				
		*p-*value	Odds Ratio			*p*-value		Odds Ratio			
			estimate	L95%	U95%			estimate	L95%	U95%	
Gender	female v male	–	–	–	–	–		–	–	–	
Ethnicity	hispanic v non-hispanic	–	–	–	–	–		–	–	–	
Race	nonwhite v white	–	–	–	–	–		–	–	–	
Relevant foreign device	yes v no	–	–	–	–	0.03		6.3	1.2	34	
Any chronic lung disease	yes v no	–	–	–	–	–		–	–	–	
Immunocompromised	yes v no	–	–	–	–	–		–	–	–	
Solid tumor	yes v no	–	–	–	–	–		–	–	–	
Diabetes mellitus	yes v no	0.03	2.1	1.1	4.3	0.006		12	2.1	70	
Age	per year	–	–	–	–	–		–	–	–	
Biofilm	per unit	–	–	–	–	–		–	–	–	
Factor 1	per unit	0.05	1.4	1.0	1.8	0.05		1.9	0.99	3.8	
Factor 2	per unit	–	–	–	–	–		–	–	–	
Factor 3	per unit	–	–	–	–	–		–	–	–	

## Discussion

In this study, we hypothesized that distinct virulence traits of the opportunistic pathogen *P. aeruginosa* would be associated with increased risk of infection vs. colonization in hospitalized patients with positive *P. aeruginosa* cultures. The presence of *P. aeruginosa* in conjunction with evidence of host tissue impairment and a local or systemic immune response was indicative of infection (Table 1), while the presence of bacteria with no or minimal tissue damage or inflammation was taken to represent colonization. We assayed a set of bacterial phenotypes, primarily chosen on the basis of their prior association with virulence in animal models of acute infection or their co-regulation with such virulence traits. Bacterial phenotypes predominantly associated with persistence in chronic infection models (e.g. production of quorum sensing molecules or pyocyanin) were not considered. Factor analysis of the measured bacterial variables revealed interrelationships between several of these phenotypes. Some groupings were expected, such as that of the five measured T3SS proteins that loaded onto Factor 1. Others were not anticipated, such as the association of twitching motility with the PAPI-1 loci RL033 and RL112 within Factor 3, but are biologically plausible given the recent finding that the PAPI-1 pathogenicity island is transferred by conjugation via a Type IV pilus encoded therein [42]. The association of two flagellar-based forms of motility with protease secretion in Factor 2 may reflect the co-regulation of both flagellar genes and the Type II secretion system by the cyclic AMP dependent transcriptional activator Vfr [43].

Most published studies report bacterial virulence phenotypes as a binary variable (i.e., present/absent). Our study differed in providing quantitative measures for most variables, and this information was retained in the statistically defined factors used in our analysis. Although studies commonly stratify risk factors when considering association with risk of disease (e.g., CD4 count and risk of opportunistic infection, LDL cholesterol and risk of coronary artery disease), this approach is not widespread in studies of microbial pathogenesis, where two-way comparisons are often made between bacteria that do or do not express a putative virulence trait.

Production and secretion of T3SS components and effectors as measured by Factor 1 was the only bacterial variable that was associated with increased risk of infection in our total cohort (odds ratio 1.4), albeit with a *p* value = 0.5. The host risk factor diabetes mellitus was also associated with an increased risk of infection (odd ratio 2.1; *p*<0.05) in this parsimonious model. The association of T3SS with clinical infection was weaker than we expected based on data from animal models of acute pulmonary infection. Indeed, in the subset of patients with respiratory tract *P. aeruginosa* isolates, no association between pneumonia and T3SS was observed. Reanalysis of the data applying a less stringent definition of infection, which allowed patients with tracheobronchitis to be classified as infected, did not result in the association of T3SS or any other bacterial variable with increased risk of infection in the multivariate or parsimonious models (data not shown). In the subset of patients with urinary tract infections, two independent parsimonious models could be constructed. The host factors of diabetes mellitus and the presence of a urinary tract catheter or stent were associated with increased risk of infection in both models, along with either bacterial T3SS protein secretion (Factor 1) or the expression of flagellar based motility and protease secretion (Factor 2). A greater propensity for protease secretion by human urinary tract isolates, as opposed to isolates from other body sites, has been reported in several studies (recently reviewed in [44]), suggesting that this group of virulence factors may play a specific role in causing disease at this anatomic site. It is not known whether decreased protease secretion by quorum-sensing mutants underlies the observed attenuation of quorum-sensing deficient *P. aeruginosa* strains in murine models of acute ascending pyelonephritis [45].

Several series have examined whether T3SS production by *P. aeruginosa* is associated with poor outcomes for human pulmonary infection. Roy-Burman et al. reported an association between increased mortality and bacterial secretion of one or more T3SS proteins in a study of 108 patients with lower respiratory tract or blood isolates of *P. aeruginosa* [11]. The interpretation of this study is confounded by the inclusion of patients with Cystic Fibrosis, whose chronic pulmonary colonization by T3SS-negative strains rarely results in severe, systemic disease. Hauser and colleagues studied a more homogeneous retrospective cohort of 35 patients with an established diagnosis of *P. aeruginosa* ventilator-associated pneumonia, and reported that infection with a T3SS-positive isolate was significantly associated with severe disease, defined as death or relapse despite appropriate therapy [10]. These findings were corroborated by a recent study demonstrating increased risk of persistence and relapse associated with T3SS-positive *P. aeruginosa* strains in patients with ventilator-associated pneumonia [9]. These latter two studies supported an association between T3SS and more severe disease in patients with ventilator-associated pneumonia, but their design did not allow them to assess the influence of this bacterial variable on the risk of infection vs. colonization in the respiratory tract (as was considered in the current study). These studies also did not consider a role for other potential virulence factors in human respiratory infection.

### Limitations and Strengths of the Current Study

Our study has limitations. First, all assays required culture and isolation of *P. aeruginosa* from primary clinical specimens, and all bacterial virulence phenotypes were measured in vitro. Although bacterial phenotypes of banked isolates were stable and reproducible upon repeat assay, we only determined whether an isolate could express a particular virulence factor under defined in vitro conditions. Thus our assays measured the potential for an isolate to express a virulence factor in the human host, but did not determine whether those virulence factors were actually expressed in the host. This limitation also applies to other studies that measure virulence factor expression of clinical isolates. Second, the definitions for infection that we applied were based on NNIS system criteria and are congruent with clinical practice. However, our definition of respiratory tract infection was rigorous in requiring radiographic findings consistent with pneumonia, and was not met by patients with tracheobronchitis; in contrast, our definition of urinary tract infection was satisfied by evidence of a local inflammatory response even in patients with a urinary catheter. Nonetheless, our findings did not change when we broadened our criteria for respiratory tract infection to include tracheobronchitis. Third, patients were followed clinically for only 5 days after the *P. aeruginosa* culture that triggered enrollment was obtained. Any individual who met criteria for infection after this time would nonetheless be considered “colonized” in our analysis.

*P. aeruginosa* causes nosocomial infections at sites beyond the respiratory tract. A strength of our study cohort is inclusion of patients with positive cultures from lung, urine and deep wound or tissue, making it more representative of patients with positive cultures for *P. aeruginosa* seen in tertiary care hospitals. Despite the heterogeneity of this patient population, we observed an increased risk of *P. aeruginosa* infection associated with T3SS-positive isolates. This association persisted in the subgroup of patients with a urinary tract source of *P. aeruginosa*, arguing that T3SS-associated virulence is not specific to the respiratory tract.

### Concluding Observations

The T3SS of *P. aeruginosa* is a dominant virulence factor in acute infections of model organisms that range from insects [46] to mice [8]. In our study, the T3SS was associated with increased risk of infection, but the magnitude of the effect was smaller than might be predicted from studies with inbred mice. Our result illustrates that in human patients, host-related variability has an equal or greater influence on the outcome of host-pathogen encounters than production of virulence factors by bacterial isolates.

Although the culture of certain organisms from clinical specimens, e.g. *M. tuberculosis*, is diagnostic of infection, the prognostic implications of culturing organisms such as *P. aeruginosa* are less clear. The identification of microbial virulence factors that influence the development of infection in humans may aid clinician decision-making in such a setting, and prompted us to undertake this study. Consideration of patient comorbidities that increase the risk and severity of infection, such as diabetes mellitus, is an integral part of treatment algorithms used by clinicians. Our findings suggest that an intrinsic bacterial factor, namely production of T3SS proteins, is also associated with an increased risk of *P. aeruginosa* infection in a heterogeneous cohort of hospitalized patients. By considering the T3SS phenotype of a clinical *P. aeruginosa* isolate, clinicians may be able to direct appropriate antimicrobial therapy to patients at higher risk of infection while still limiting the indiscriminate use of antimicrobials associated with increased antibiotic resistance [47]. Our findings also support further investigation into therapeutic strategies that target the T3SS and its effectors [48]–[52].

## Supporting Information

Text S1 **Equations determined by factor analysis.**
(DOCX)
