# Supplementary material for: The Ability of Virulence Factor Expression by Pseudomonas aeruginosa to Predict Clinical Disease in Hospitalized Patients
Source: PLoS One. 2012 Nov 12;7(11):e49578. doi: 10.1371/journal.pone.0049578 (PMC3495863; doi:10.1371/journal.pone.0049578)
Supplement: Text S1 — Equations determined by factor analysis. (DOCX) [file pone.0049578.s001.docx]

**Figure S1. Equations determined by factor analysis**

**Factor 1**: **0.17180 * (ExoU) + 0.17472 * (ExoS) + 0.28156 * (ExoT) + 0.27342 * (PcrV) + 0.28468 * (PopD)** - 0.00985 * (swimming) + 0.01218 * (swarming) + 0.03658 * (twitching) - 0.00661 * (proteases) - 0.00296 * (RL033) - 0.00422 * (RL112)

**Factor 2**: -0.13468 * (ExoU) + 0.07836 * (ExoS) - 0.03907 * (ExoT) + 0.05225 * (PcrV) + 0.00227 * (PopD) **+ 0.35596 * (swimming) + 0.37548 * (swarming)** + 0.15030 * (twitching) **+ 0.42199 * (proteases)** - 0.17478 * (RL033) - 0.00313 * (RL112)

**Factor 3**: -0.26189 * (ExoU) + 0.18344 * (ExoS) - 0.03039 * (ExoT) + 0.05511 * (PcrV) - 0.01633 * (PopD) + 0.02914 * (swimming) - 0.14786 * (swarming) **+ 0.33490 * (twitching)** + 0.12291 * (proteases) **+ 0.42844 * (RL033) + 0.48969 * (RL112)**
